# The complete mitochondrial genome of *Conwentzia sinica* (Neuroptera: Coniopterygidae)

**DOI:** 10.1080/23802359.2019.1688714

**Published:** 2019-11-13

**Authors:** Jialu Song, Jiayu Dong, Mingwei Ma, He Yi, Zhiqi Liu

**Affiliations:** Department of Entomology, China Agricultural University, Beijing, China

**Keywords:** Coniopterygidae, *Conwentzia sinica*, mitochondrial genome

## Abstract

The complete mitochondrial genome (mitogenome) of *Conwentzia sinica* Yang, 1974 was sequenced and analyzed. The sequenced mitogenome is 15,153 bp in size, including 13 protein-coding genes (PCGs), 22 tRNA genes, 2 rRNA genes, and one control region. Thirteen PCGs use ATN as the start codon. Most of PCGs terminate with TAA codons but *ND5*, *ND4* with a single T residue, and *ND3* terminates with TAG. The *lrRNA* gene is 1290 bp. The measured length of *srRNA* gene is 782 bp. Twenty-two tRNA genes possess the typical clover-leaf secondary structure except for *tRNA^Ser(AGN)^*. The phylogenetic result supports the monophyly of the family Coniopterygidae and a closer relationship between *Conwentzia* and *Coniopteryx*.

*Conwentzia* Enderlein, 1905 belongs to family Coniopterygidae, which is the smallest and most special group in the order Neuroptera (Wang and Liu 2007). *Conwentzia sinica* Yang, 1974 is an important predatory natural enemy of *Oligonychus perditus* Pritchard and Baker, 1955 (Xu et al. 2007). However, the phylogenetic relationship within the family Coniopterygidae from the perspective of molecular biology is still almost blank (Wang and Liu 2007). In this study, the complete mitochondrial genome (mitogenome) of *C. sinica* was reported and the phylogenetic relationship was reconstructed combining the current mitogenome data of other two Coniopterygidae species and other six families of Neuroptera.

In this study, the specimen was obtained from Penglai Park in Shanghai, China. Voucher specimen (No. CAU-Cwen-SH2) was deposited at the Entomological Museum of China Agricultural University (CAU). The mitogenome was sequenced by next-generation sequencing method with Illumina Hiseq 2500 (Sangon Biotech, Beijing) and the sequence was deposited in GenBank under the MN200022.

The sequenced mitogenome is 15,153 bp in length, including 13 protein-coding genes (PCGs), 22 tRNA genes, two rRNA genes, and one control region. The base composition is: A, 40.6%; T, 38.3%; C, 12.1%; G, 9.0%. It showed that the nucleotide composition of the mitochondrial genome is significantly biased towards A + T (78.9%) with the negative AT-skew 0.028) and negative GC-skew (−0.149). The gene arrangement is consistent with the putative ancestor of insects and also other two Coniopterygidae species, e.g. *Semidalis aleyrodiformis* and *Coniopteryx* sp. (Wang et al. 2017). All PCGs start with ATT codon except for *COII*, *ND3*, *ND5*, *ND1* (ATA), *ATP6*, *COIII*, *ND4*, *CytB* (ATG). All of the PCGs terminate with the complete stop codon (TAA for *ND2*, *COI*, *ATP8*, *ATP6*, *COIII*, *ND4L*, *ND6*, *ND3*, *CytB*, *ND1*; *TAG* for ND3) except *ND4* and *ND5* with a single T residue as the termination codon. The *lrRNA* is 1290 bp in length with an A + T content of 84.2% and the *srRNA* is 782 bp in length with an A + T content of 83.7%. The control region located between the *srRNA* and *tRNA^Ile^* is 416 bp with A + T content of 90.2%. The length of 22 *tRNA* genes measured ranged from 56 bp to 71 bp. All *tRNA* genes possess the typical clover-leaf secondary structure except for *tRNA^Ser(AGN)^*, which lacks a dihydroxyuridine (DHU) arm.

The phylogenetic tree was constructed based on the sequences of 13 protein-coding genes from 10 neuropteran species by using Phylobayes MPI on XSEDE (1.5a) (Lartillot et al. 2009) with method under the GTR + CAT model (Song et al. 2016; Li et al. 2017; Liu et al. 2018). The result supports the monophyly of the family Coniopterygidae and shows *Coniopteryx* has a closer relationship with *Conwentzia* than with *Semidalis*. This result about the relationship of the three genera is different from the morphological classification in Coniopterygidae, therefore, further research is needed (Figure 1).

**Figure 1.**
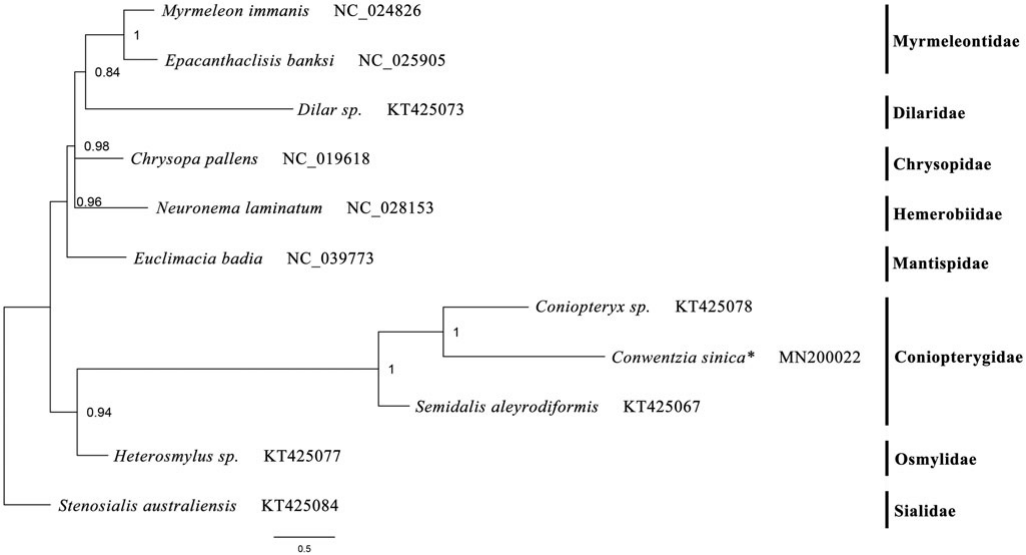
Phylogenetic tree inferred from analysis of the nucleotide of the 13 PCGs. The nodal numbers indicate the Bayesian posterior probability. GenBank accession numbers for the sequences are indicated next to the species names. The newly sequenced mitogenome is indicated by the asterisk.
